# The Stability of Phyto-Zooplanktonic Networks Varied with Zooplanktonic Sizes in Chinese Coastal Ecosystem

**DOI:** 10.1128/msystems.00821-22

**Published:** 2022-10-06

**Authors:** Zheng Zhang, Hongjun Li, Wenli Shen, Kai Feng, Shuzhen Li, Songsong Gu, Yuqi Zhou, Xi Peng, Xiongfeng Du, Qing He, Linlin Wang, Zhaojing Zhang, Danrui Wang, Zhujun Wang, Ye Deng

**Affiliations:** a Institute of Marine Science and Technology, Shandong University, Qingdao, China; b State Environmental Protection Key Laboratory of Coastal Ecosystem, National Marine Environmental Monitoring Center, Dalian, China; c CAS Key Laboratory for Environmental Biotechnology, Research Center for Eco-Environmental Sciences, Chinese Academy of Sciences, Beijing, China; d College of Resources and Environment, University of Chinese Academy of Sciences, Beijing, China; e Aquatic EcoHealth Group, Fujian Key Laboratory of Watershed Ecology, Key Laboratory of Urban Environment and Health, Institute of Urban Environment, Chinese Academy of Sciences, Xiamen, China; California State University, Northridge

**Keywords:** zooplankton, body size, phyto-zooplanktonic network, network stability, coastal ecosystem

## Abstract

The linkages between phytoplankton and zooplankton are crucial for the stability of complex food webs and the flow of energy within the marine ecosystem. Despite body size exhibiting multiple effects on the planktonic community assembly and the dispersal scale, its role in determining the stability of phyto-zooplanktonic co-occurrence patterns remains unclear. Here, we focused on more than 13,000 kilometers of the Chinese coast to study the diatom-dominated plankton ecosystem and to report the significant negative effects of zooplanktonic body sizes on the topological properties of phyto-zooplanktonic networks (PZNs) by using more than 500 species from 251 samples taken along the coastline. PZNs tended to be more complex and stable when phytoplankton associated with smaller zooplankton. Particularly, the subnetworks of dominant phytoplankton displayed differences with different zooplanktonic body sizes. The zooplankton with larger and smaller body sizes tended to interact with dinoflagellates and diatoms, respectively. Additionally, abiotic factors (i.e., water temperature, pH, salinity, and metal concentrations) displayed significant effects on PZNs via the shifting of zooplanktonic composition, and the zooplanktonic body sizes altered the network modules’ associations with different environmental factors. Our study elucidated the general relationship between zooplanktonic body sizes and the stability of PZNs, which provides new insights into marine food webs.

**IMPORTANCE** Body size is a key life trait of aquatic plankton that affects organisms’ metabolic rates and ecological functions; however, its specific effects on interactions between phytoplankton and zooplankton are poorly understood. We collected planktonic species and their body size data along more than 13,000 kilometers of coastline to explore the role of zooplanktonic body size in maintaining the stability of phyto-zooplanktonic networks (PZNs). We found that zooplankton play a more important role in maintaining PZN stability than do phytoplankton as well as that the PZN would be more complex and stable with smaller zooplankton. Furthermore, this work revealed that body size significantly determined the relationships between environmental factors and network structure. Overall, these findings lay a general relationship between zooplanktonic body sizes and the stability of PZNs, which helps us further explore the micro food web of coastal ecosystems.

## INTRODUCTION

The ocean is teeming with microsize organisms, including viruses, bacteria, phytoplankton, zooplankton, and the larvae of fishes (1). This planktonic diversity is extremely complex and drives the marine carbon cycle (2). Phytoplankton form the bases of food webs and are the main energy sources for aquatic ecosystems (3, 4). Diatoms are the most commonly observed phytoplanktonic group in aquatic ecosystems, accounting for approximately 23% of the primary production and playing a key role in the structures of the food web and carbon cycle (5, 6). Another dominant taxon, dinoflagellates, are mainly distributed in ocean surface waters (almost 90%), and their frequent outbreaks negatively affect aquatic ecosystems through the production of “red tides” and other “harmful algal blooms” (7). In parallel, marine zooplankton occupy multiple trophic levels in aquatic food webs and could alter the pelagic food web through their respective trophic interactions with the primary producers and fishes that are above and below them, as well as through the transfer of particulate carbon into the dissolved carbon pool (8). Although the micro food web formed by the interaction between phytoplankton and zooplankton is the core structuring ecosystem function, carbon exporter, and regulator of climate variation (9, 10), we currently lack a clear understanding of the stability of phyto-zooplankton interactions in natural marine ecosystems, especially at a large-scale.

The body size of an organism is one of the important physiological and functional traits that affect individual metabolic rates and the stability of food webs, as, for example, smaller organisms have higher metabolic rates (11–14). Body size may shape the interactions between zooplankton and phytoplankton by determining zooplanktonic feeding efficiency and the energy transmission efficiency of the microscale food web (15). Previous studies have indicated that the interactions between smaller organisms (primary producers) and larger organisms (e.g., grazers, copepods) determine ecosystem stability (16) and have found that body size is one of the key traits that could directly determine microbial distance-decay patterns (17). Additionally, most zooplanktonic species possess a long larval stage with a small body size (18), and these factors can be beneficial to its dispersal in aquatic ecosystems (19). Such studies have focused on the size variation between- taxonomic groups, but, so far, few studies have investigated the impact of within-group body size on biotic interactions (14). Considering the importance of zooplanktonic taxa in linking the aquatic food web and the major role of body size in structuring microbial communities, exploring how zooplanktonic size variation impacts phyto-zooplankton relationships is critical for understanding the marine plankton ecosystem and managing coastal ecological stability.

Ecological networks have been widely used to investigate co-occurrence patterns among microorganisms in marine and soil environments (20, 21). Although network analysis-based approaches have limitations in distinguishing the mechanisms of complex systems, they are still essential for predicting potential microbial organism interrelationships and community organization (22–28). In our previous studies, cross-trophic relationships were explored using bipartite networks, including plant-microbe associations and cercozoan-prokaryote interaction patterns, to reveal the microbial indicators that respond to warming and interdomain species associations (21, 29). In addition, using network analysis as an exploratory tool, many studies have indicated that interactions between microbial organisms were the main driving force in maintaining community stability (30–33). Ecological stability indicates the capacity of microbial communities to resist climate variation and recover from disturbances, which might be reflected by the topological properties and complexity of the microbial networks (34, 35). For example, a microbial network with higher complexity showed higher stability under warming (36), while a network with a lower modularity and a lower ratio of negative-to-positive cohesion displayed unstable properties under stress (37). Previous studies have demonstrated that the keystone organisms played crucial roles in maintaining network stability, such that microbial stability increased with the number of hub taxa (38, 39). Additionally, based on network theory, higher robustness and lower vulnerability indicate a more stable network (25, 31, 36, 40). However, the effect of body size on phyto-zooplankton interactions in aquatic ecosystems, which are crucial for understanding the coastal ecosystem stability, remain unclear.

In this study, a total of 251 seawater samples from 12 different areas were collected, covering 13,000 km of Chinese coastline between 20°N and 40°N. Our previous study found a clear latitudinal pattern for phytoplanktonic and zooplanktonic diversity as well as the phyto-zooplankton interaction network, but the effect of zooplanktonic body size on PZNs stability has not been sufficiently explored, relative to the geographic patterns of diversity and interactions (41). Here, we constructed separate PZNs for 12 sampling sites to test the effects of zooplanktonic body size on the stability of PZNs. Importantly, the current study separated the zooplanktonic species into two size groups (~160 μm and ~500 μm) based on the mesh size of the plankton net, and only those species observed in both body size fractions were retained for subsequent analysis. Considering the higher metabolic rate and environmental sensitivity of smaller-sized organisms, we proposed the following two hypotheses: (i) zooplanktonic species with smaller body sizes tend to construct more stable interaction networks with phytoplankton in coastal ecosystems; (ii) the variation of zooplanktonic body size could alter the response of the planktonic community to environmental changes.

## RESULTS

In order to eliminate the interference due to variations in zooplanktonic community composition, we removed species that were collected alone by the I (40 species) and II (82 species) planktonic net, and only the shared species of smaller and bigger zooplankton were retained. This resulted in a total of 200 zooplanktonic species, which could be classified into 15 phyla, 25 classes, and 125 genera based on the World Register of Marine Species (WoRMS, http://www.marinespecies.org/). In general, zooplanktonic density declined significantly with the increase in body size (Wilcoxon test, *P* < 0.01).

### Bipartite PZNs of different zooplanktonic sizes.

**FIG 1:**
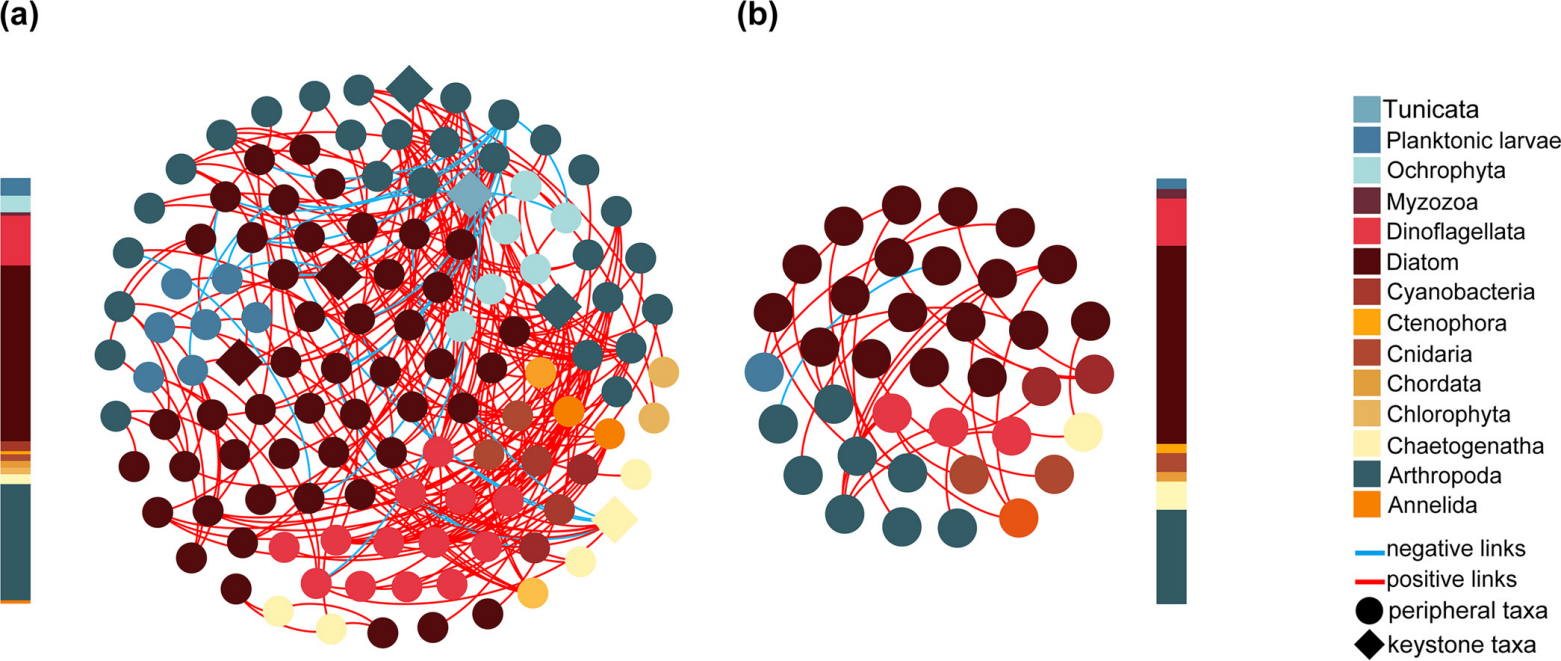
Co-occurrence network of small size zooplankton (a) and big size zooplankton (b) interactions with phytoplankton. The networks are colored based on the planktonic taxa. Blue and red links represent significantly negative and positive correlations. The bar of each network shows the proportions of the networked taxa.

To characterize variations of the phyto-zooplankton networks (PZNs) with zooplanktonic body size, sparse correlations for compositional data (SparCC)-based co-occurrence networks were constructed. The plankton cross-trophic networks consisted of 45 and 128 planktonic species (nodes) with 40 and 295 edges in the phytoplankton-bigger zooplankton interaction network (PBZN) and the phytoplankton-smaller zooplankton network (PSZN), respectively (Fig. 1). In addition, we also generated bipartite networks between smaller and bigger zooplankton, and we found that all links were positive (Fig. S1B). The networked zooplanktonic density was measured and found to be significantly higher in the PSZNs than in the PBZNs (Fig. S2A). Networked smaller and bigger zooplankton community relationships were visualized via nonmetric multidimensional scaling (NMDS), and a significant difference was observed using a multiple response permutation procedure (MRPP) (δ = 0.95, *P* = 0.009), an analysis of similarities (ANOSIM) (*R* = 0.11, *P* = 0.037), and a nonparametric multivariate analysis of variance (PERMANOVA) (F = 1.99, *P* = 0.002) (Fig. S2B). The composition of networked taxa was different between the networks. Most networked nodes were dominated by anthropods, diatoms, and dinoflagellates, but the relative abundance of Ochrophyta and Chaetognatha were decreased and increased, respectively, with the increase in networked zooplanktonic body size (Fig. 1). These results showed that the networked zooplankton composition varied significantly with the larger zooplankton size fraction.

10.1128/msystems.00821-22.1 FIG S1 (a) Map of the Chinese coastline indicating the sampling sites. (b) Visualization of the constructed bigger zooplankton and smaller zooplankton co-occurrence network. The network was colored by the zooplankton composition, and the large and small nodes represent the big and small size zooplankton species, respectively. Red links represent significant positive correlations. Download FIG S1, TIF file, 1.3 MB. Copyright © 2022 Zhang et al. 2022 Zhang et al. https://creativecommons.org/licenses/by/4.0/ This content is distributed under the terms of the Creative Commons Attribution 4.0 International license.

10.1128/msystems.00821-22.2 FIG S2 The networked species diversity and the community structure driving factors. (A) The density of the networked zooplanktonic species (bigger zooplankton, smaller zooplankton, and phytoplankton). (B) Nonmetric multidimensional scaling (NMDS) of the structure of the networked zooplanktonic communities. The clusters of the zooplankton community were confirmed by a permutational multivariate analysis of variance (ADONIS), a multiple response permutation procedure (MRPP), and a similarity analysis (ANOSIM). (C) Canonical correspondence analysis (CCA) of the links between the networked planktonic community structure and environmental drivers. (D) Variation partitioning analysis (VPA) separating the variation of community structure explained by the CCA model. Temp, temperature; water traits: temperature, salinity, and dissolved oxygen [DO]; artificial stresses: NO_2_-N, COD, As, Pb, NH_4_-N, and Zn; and distance (PCNM). Download FIG S2, TIF file, 1.8 MB. Copyright © 2022 Zhang et al. 2022 Zhang et al. https://creativecommons.org/licenses/by/4.0/ This content is distributed under the terms of the Creative Commons Attribution 4.0 International license.

We also examined the influence of environmental and spatial factors on shaping the networked community compositions. Distance (principal coordinates of neighborhood matrix [PCNM]), NO_2_-N, NH_4_-N, and temperature played significant but weak roles in shaping the structure of the networked plankton communities, as revealed by a canonical correlation analysis (CCA), and the water temperature was the most important factor that significantly shaped the networked planktonic community heterogeneity (model is significant with *P* = 0.001 tested by ANOVA) (Fig. S2C; Table S1). The contribution of water traits (temperature, salinity, and dissolved oxygen [DO]), artificial stresses (NO_2_-N, COD, As, Pb, NH_4_-N, and Zn), and distance (PCNM) to networked communities was illustrated with a variation portioning analysis (VPA), and it was found that the networked community was only weakly affected by those abiotic factors, which only explained 26.75% of the variation (Fig. S2D). The majority of the unexplained variation may be due to unmeasured environmental variables and drift. The above results showed that the networked zooplankton community composition was significantly different with body size. In order to determine the effect of zooplanktonic body size on the complexity of PZNs, changes of network topological parameters were examined. The PSZN showed higher network topology values compared to those of PBZN, including the network size, negative correlation links, links per species, cluster coefficient, linkage density, and weighted connectance (Table 1). Also, the empirical networks were significantly different from those of the corresponding random networks, indicating that nonrandom features of the observed topological structures of PZNs as well as the PZN complexity feature decreased with increased zooplanktonic body size.

**TABLE 1:** Global network properties of phyto-zooplankton interaction networks

		Empirical networks							Random networks		
Phyto-zooplankton network^*b*^	Networked taxa	Network size	Links	Negative links	Links per species	Cluster coefficient	Linkage density	Weighted connectance	Cluster coefficient	Linkage density	Weighted connectance
PBZN	Zooplankton	19	40	5%	0.889	0.0526^*a*^	2.375^*a*^	0.053^*a*^	0.067 (4.18 × 10^−17^)	2.29 (2.23 × 10^−15^)	0.062 (4.28 × 10^−17^)
	Phytoplankton	26									
PSZN	Zooplankton	50	295	10.17%	2.306	0.06^*a*^	7.929^*a*^	0.062^*a*^	0.039 (5.58 × 10^−17^)	6.26 (8.93 × 10^−16^)	0.051 (1.12 × 10^−16^)
	Phytoplankton	78									

a Significant difference between empirical and randomized networks.

b PBZN, phytoplankton-bigger zooplankton interaction network; PSZN, phytoplankton-smaller zooplankton interaction network. The random network values are means (standard deviations) based on the results of 1,000 rewiring networks.

10.1128/msystems.00821-22.8 TABLE S1 Analysis of variance (ANOVA) of environmental factors and distance correlated with networked planktonic beta diversity. Statistical significance is indicated by numbers in bold. Download Table S1, DOCX file, 0.02 MB. Copyright © 2022 Zhang et al. 2022 Zhang et al. https://creativecommons.org/licenses/by/4.0/ This content is distributed under the terms of the Creative Commons Attribution 4.0 International license.

### The stability of PZNs with different zooplanktonic size fractions.

**FIG 2:**
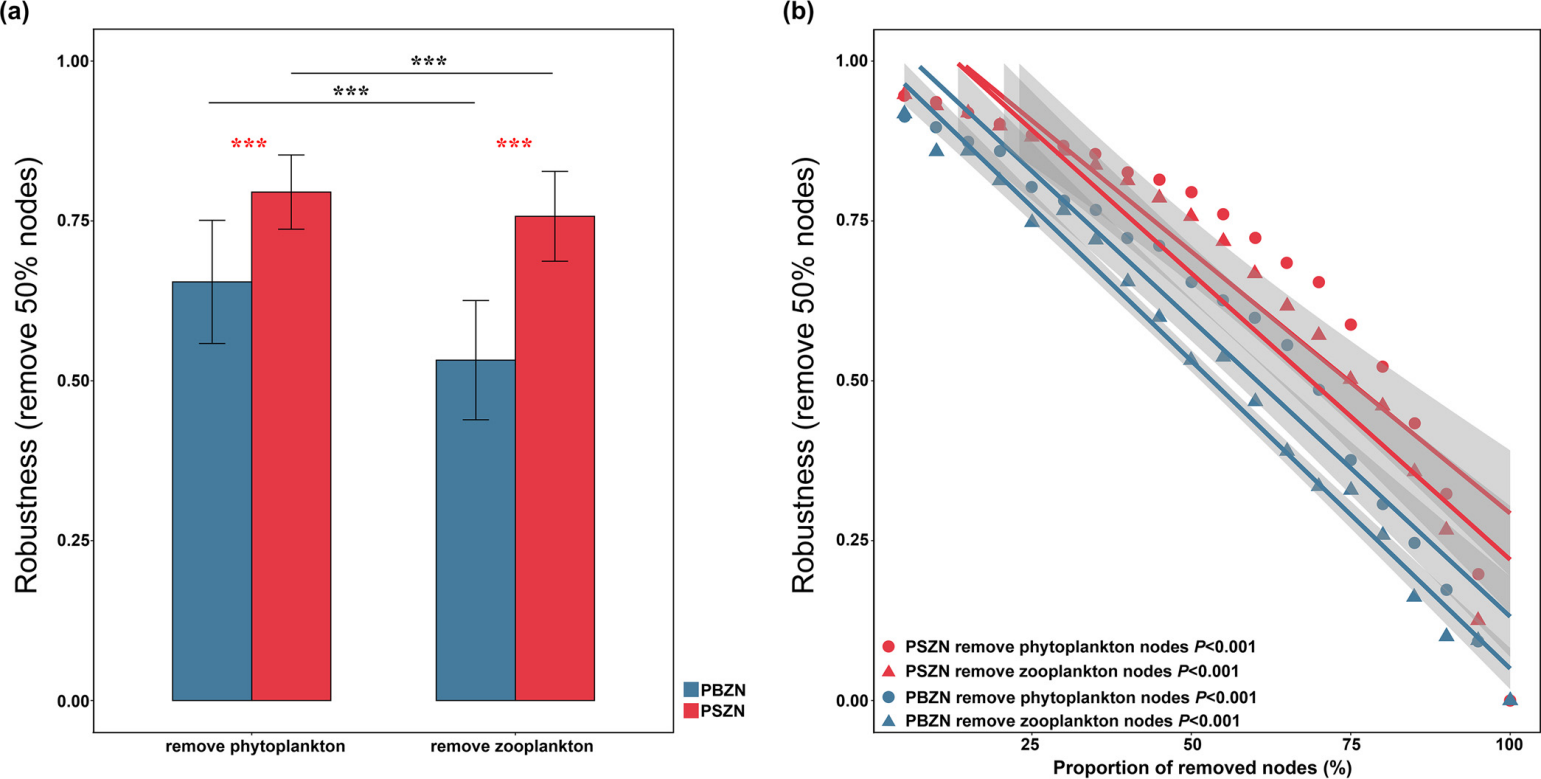
The resistance of PZNs to node loss. (a) The robustness of PBZN and PSZN were defined as the proportions of the remaining species in this network after randomly removing 50% of the zooplanktonic or phytoplanktonic nodes. Error bars correspond to the standard deviation, and asterisks indicate the statistical significance of the correlation (***, *P* < 0.001; two-sided *t* test). (b) Relationships between planktonic network robustness and the proportion of removed nodes were estimated via linear least-squares regression analysis. A higher slope represents a more drastic decline in network structural robustness. The fitting lines are shown with their 95% confidence regions.

To explore whether the stability of the PZNs changed with zooplanktonic body size, the resistance of all of the PZNs to node loss (robustness) was calculated. To confirm the observed network robustness, the rewiring approach was used to generate 1,000 random bipartite networks, and the robustness was compared for the observed PZNs with their corresponding rewired networks (Fig. S3A). First, on the basis of either random zooplanktonic or phytoplanktonic species loss, the random PSZNs had significantly higher robustness values than did the PSZNs. Additionally, significantly higher robustness values were observed for the random PBZNs when the zooplanktonic species were randomly removed than were observed for the PBSZ. The above results suggest nonrandom features of the robustness of the observed PZNs. Next, we found that the PSZN exhibited significantly higher robustness to either randomly removed zooplanktonic or phytoplanktonic nodes (Fig. 2A) and that as the proportion of removed nodes increased, the robustness was also, on average, higher in the PSZN than in the PBZN (Permutation test = 999, *P* < 0.001) (Fig. 2B). These results indicated that a more stable PZN was constructed by smaller zooplankton. In addition, the robustness was consistently higher when randomly removing phytoplanktonic nodes than when removing zooplanktonic nodes, suggesting that the zooplanktonic species occupied a more central position in determining network stability than did the phytoplankton (Fig. 2A).

10.1128/msystems.00821-22.3 FIG S3 Robustness was measured as the proportion of taxa remaining with 50% of the different trophic taxa randomly removed from each of the observed and random entire PZNs (A) and regional PZNs (B and C). Error bars correspond to the standard deviations, and asterisks indicate the statistical significance of the correlation (***, *P* < 0.001; two-sided *t* test). Download FIG S3, TIF file, 1.7 MB. Copyright © 2022 Zhang et al. 2022 Zhang et al. https://creativecommons.org/licenses/by/4.0/ This content is distributed under the terms of the Creative Commons Attribution 4.0 International license.

In order to further reveal the pattern of zooplanktonic body size on PZNs stability, we constructed PZNs for the 12 sites, individually (Fig. S4). Although the number of small and big size zooplankton species used in constructing the PZN for each site was equal, the network size and links for most PSZNs (83.33%) were higher than those of the PBZNs, suggesting that the phytoplankton tended to be more interconnected with smaller sized zooplankton (Fig. S4; Table S2).

10.1128/msystems.00821-22.4 FIG S4 Regional PBZNs and PSZNs along the coastline. The node color indicates the planktonic taxon. Blue and red links represent significantly negative and positive correlations, respectively, and the proportions of the link types are shown by bars beside the corresponding PZNs. Download FIG S4, TIF file, 1.9 MB. Copyright © 2022 Zhang et al. 2022 Zhang et al. https://creativecommons.org/licenses/by/4.0/ This content is distributed under the terms of the Creative Commons Attribution 4.0 International license.

10.1128/msystems.00821-22.9 TABLE S2 Topological properties of the PZNs with different sized zooplankton along the Chinese coastline. Download Table S2, DOCX file, 0.02 MB. Copyright © 2022 Zhang et al. 2022 Zhang et al. https://creativecommons.org/licenses/by/4.0/ This content is distributed under the terms of the Creative Commons Attribution 4.0 International license.

**FIG 3:**
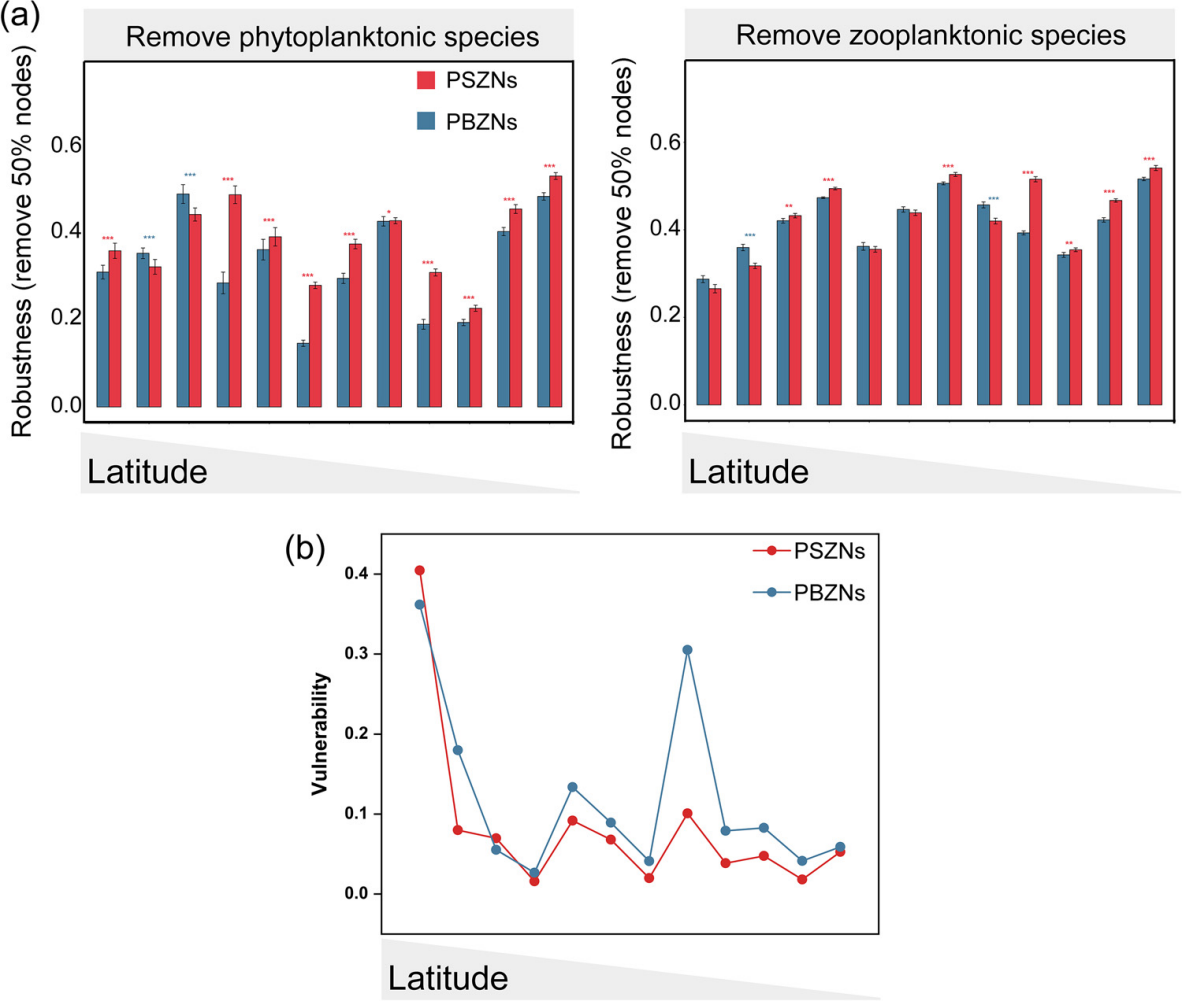
(a) The structural robustness of the regional PBZNs and PSZNs, as assessed by the proportion of the remaining species in this network after randomly removing 50% zooplanktonic or phytoplanktonic nodes. The error bars correspond to the standard deviation, and the asterisks represent the statistical significance of the correlation (***, *P* < 0.001; *, *P* < 0.05; two-sided *t* test). (b) Network vulnerability, as measured by the maximum node vulnerability of each network, for the PBZNs and PSZNs at each site.

Significantly higher robustness values were observed in most regional PSZNs (66.67%) when phytoplanktonic nodes were randomly removed, and only 16.67% of PBZNs exhibited significantly higher robustness values than did the PSZNs when zooplanktonic nodes were removed (Fig. 3A). Additionally, we calculated the resistance to zooplanktonic and phytoplanktonic node loss of each 1,000 random regional PZNs, respectively, and we found that the robustness values of all of the random PZNs were significantly different from those of the observed PZNs (*t* test, *P* < 0.05), indicating that the PZNs with different zooplanktonic body sizes exhibited nonrandom stable features (Fig. S3B and C). The network vulnerability comparison between each regional PSZN and PBZN showed that the index was lower overall (83.33%) in the PZNs with smaller body size zooplankton (Fig. 3B). Based on the within-module connectivity (*Zi*) and among-module connectivity (*Pi*), the networked nodes were classified into hub taxa (module, connector, and network hubs) and peripherals. In the entire PSZN, six planktonic taxa (Diatom, Chaetognatha, Arthropoda, and Tunicata) were detected as potential hub taxa, and no hub species were identified in PBZN (Fig. 1). Subsequently, we detected a total of 138 and 112 hub taxa across all of the regional PSZNs and PBZNs, respectively (Fig. S5), and found that relatively few zooplankton taxa retained their positions as keystone taxa in the PSZNs and PBZNs. For instance, Gastropoda was only identified as a hub in the PSZNs, while the PBZNs hubs were mainly from the genera *Acartia* and *Labidocera*, suggesting that the roles played by these differently sized zooplankton taxa differed in maintaining PZN structure and stability. Our results not only indicated that the zooplankton species played a more important role in maintaining PZN stability but also pointed out that zooplanktonic body size, as a crucial trait, exhibited a significant negative relationship with PZN stability.

10.1128/msystems.00821-22.5 FIG S5 The nodes were classified into keystone and peripheral species based on their within-module connectivity (*Zi*) and among-module connectivity (*Pi*). The red and blue represent keystone nodes, and the grey represents peripheral nodes. Download FIG S5, TIF file, 0.9 MB. Copyright © 2022 Zhang et al. 2022 Zhang et al. https://creativecommons.org/licenses/by/4.0/ This content is distributed under the terms of the Creative Commons Attribution 4.0 International license.

### Influence of zooplanktonic size on the stability of dominant phytoplankton subnetworks.

To further explore the relationship between zooplanktonic body sizes and phyto-zooplankton interactions, subnetworks of the dominant phytoplankton groups (diatoms and dinoflagellates) were extracted from all of the PZNs, respectively, and the vulnerability and robustness values were calculated. Most diatom-bigger zooplankton subnetworks (Dia&BZNs) (80%) were found to have higher vulnerability values than diatom-smaller zooplankton subnetworks (Dia&SZNs) (Fig. S6A). However, dinoflagellate-zooplankton subnetworks exhibited the opposite trend, and higher vulnerability values were found in more dinoflagellate-smaller zooplankton subnetworks (Din&SZNs) (85.71%) than in dinoflagellate-bigger zooplankton subnetworks (Din&BZNs) (Fig. S6C). For the diatom subnetworks, no difference was observed when either the phytoplanktonic or zooplanktonic nodes were removed, with more PSZNs exhibiting significantly higher robustness values than did the PBZNs (40% versus 20% and 60% versus 10%) (Fig. S6B). The robustness comparison between the dinoflagellate subnetworks at the sampling sites showed that although randomly removed zooplanktonic nodes caused most Din&SZNs to exhibit higher robustness, a majority of Din&BZNs (71.43%) were more robust than the Din&SZNs when phytoplanktonic species were lost (Fig. S6D). The robustness values of the random diatom and dinoflagellate subnetworks were significantly different from those of the observed networks, and more stable interaction networks were found in Dia&BZNs and Din&SZNs. These results demonstrated that zooplankton with different sizes might exhibit different robustness relationships with specific dominant phytoplankton.

10.1128/msystems.00821-22.6 FIG S6 The maximum node vulnerability in most diatom (A) and dinoflagellate (C) subnetworks. Subnetworks with <20 nodes were not shown. Robustness is measured as the proportion of taxa remaining with 50% of the planktonic taxa randomly removed from the subnetworks of zooplankton interacting with diatoms (B) and dinoflagellates (D). The error bars correspond to the standard deviations, and the asterisks represent the statistical significance of the correlation (***, *P* < 0.001; **, *P* < 0.01; *, *P* < 0.05; two-sided *t* test). Download FIG S6, TIF file, 2.2 MB. Copyright © 2022 Zhang et al. 2022 Zhang et al. https://creativecommons.org/licenses/by/4.0/ This content is distributed under the terms of the Creative Commons Attribution 4.0 International license.

### Linking environmental factors and the topological indices of PZNs.

Mantel tests were performed to understand the relationships between abiotic factors, planktonic community composition (Table S3), and PZNs lineages (Table 2). The phytoplanktonic community composition was less sensitive to disturbances of environmental properties than was the zooplanktonic community. The water traits (temperature, pH, and salinity) and NO_3_-N were significantly responsible for changes in the phytoplanktonic community. Besides these factors, certain metal concentrations (Hg, Pb, As, and Zn) significantly explained the variation in zooplanktonic composition with small body size. It was noted that the larger sized zooplankton fraction was more strongly correlated with the environmental dissimilarity matrix (significantly associated with all environmental variables except NO_2_-N), compared with that of the smaller organisms. Our results showed that the connectivity of zooplanktonic species with small and big sizes were significantly correlated with pH (*R *= 0.183, *P* = 0.043) and metal concentrations (*R *= 0.257, *P* = 0.045), respectively (Table 2). In addition, the phytoplanktonic connectivity in both PSZNs and PBZNs was significantly explained by the metal concentrations. This indicated that phytoplankton were less susceptible to environmental fluctuations and that zooplankton of different size fractions respond differently.

**TABLE 2:** The Mantel tests on connectivity versus the networked species significances of environmental factors in PZNs^*a*^

		Temperature^*b*^		Salinity		N		pH		Metal	
Phyto-zooplankton network	Networked taxa	*P*	R	*P*	R	*P*	R	*P*	R	*P*	R
PSZN	Zooplankton	0.857	−0.079	0.829	−0.081	0.644	−0.056	0.043*	0.183	0.573	−0.031
	Phytoplankton	0.350	0.011	0.420	−0.006	0.778	−0.063	0.267	0.034	0.001***	0.411
PBZN	Zooplankton	0.713	−0.091	0.34	−0.038	0.687	−0.107	0.282	−0.030	0.045*	0.257
	Phytoplankton	0.461	−0.044	0.379	−0.026	0.609	−0.103	0.674	−0.117	0.005**	0.721

a Statistical significance is shown by asterisks (***, *P* < 0.001; **, *P* < 0.01; *, *P* < 0.05).

b Temperature, water temperature; N, nitrate nitrogen, nitrite nitrogen, and ammonia nitrogen; Metal, the concentrations of Zn, Pb, As, Cu, and Hg.

10.1128/msystems.00821-22.10 TABLE S3 Mantel tests on the significance of environmental factors and planktonic community composition. Statistical significance is indicated by numbers in bold. Download Table S3, DOCX file, 0.02 MB. Copyright © 2022 Zhang et al. 2022 Zhang et al. https://creativecommons.org/licenses/by/4.0/ This content is distributed under the terms of the Creative Commons Attribution 4.0 International license.

Structural equation modeling (SEM) was used to reveal the relationships between environmental factors (water traits, metal concentrations, and nitrogen), planktonic composition, and network structure. The SEM revealed that 73% of the variance in the PSZNs and in the network structure could be explained by environmental factors (represented by the first component of a principal components analysis [PCA]) and the planktonic composition (represented by nonmetric multidimensional scaling 1). Metal concentrations and nitrogen had directly negative and positive effects on PSZN, respectively. The water traits did not directly link to PSZN, but they did have a direct connection to zooplanktonic composition. Notably, zooplanktonic and phytoplanktonic compositions were the most important direct predictors for PSZN (Fig. S7A). Interestingly, the statistically significant direct or indirect effects of environmental factors on the network were not the same for the different zooplanktonic size fractions, as evidenced by the fact that all relationships were not significant for PBZN (Fig. S7B). These results revealed that the environmental factors mainly affected the network structure by directly impacting the zooplanktonic composition and the crucial role of zooplanktonic body size in determining the relationship between environmental factors and PZNs.

10.1128/msystems.00821-22.7 FIG S7 Structural equation model showing the paths of environmental factors and planktonic composition modeling to (A) PSZN and (B) PBZN. Solid arrows indicate significant effect sizes (*P* < 0.05), and dashed lines represent insignificant effect sizes (*P* > 0.05). The green and red colors of the paths indicate negative and positive relationships, respectively. The width of the arrow represents the strength of the relationship. Download FIG S7, TIF file, 0.3 MB. Copyright © 2022 Zhang et al. 2022 Zhang et al. https://creativecommons.org/licenses/by/4.0/ This content is distributed under the terms of the Creative Commons Attribution 4.0 International license.

**FIG 4:**
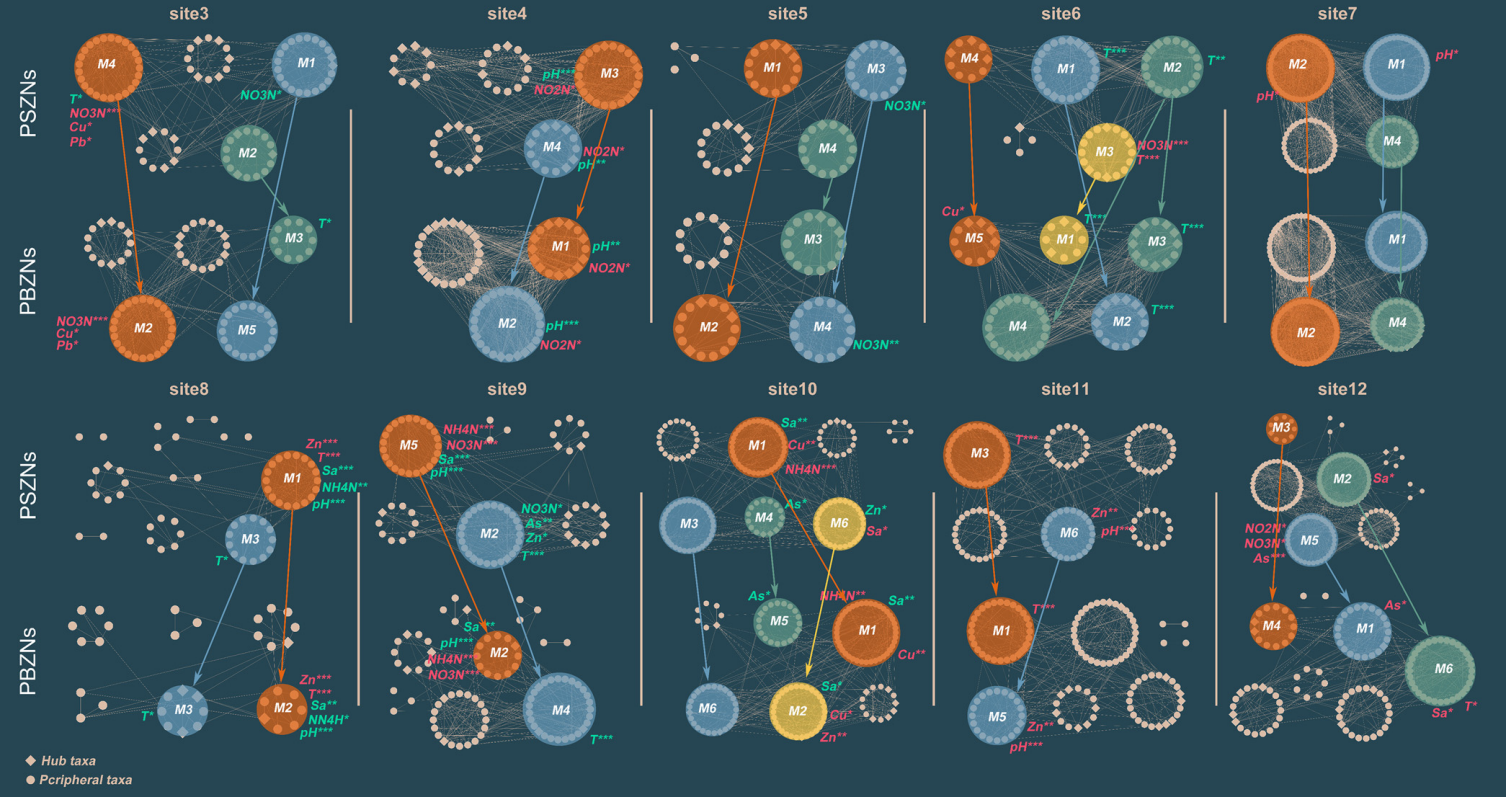
Visualization of constructed PSZNs and PBZNs for each sampling site along latitude (except for site1 and site2, in which the modules had ≤5 nodes). Modules of the same color within the same site contained a large proportion of shared nodes between the PSZNs and PBZNs, and they were regarded as preserved module pairs. These are also indicated by the arrow pointing from the PSZN to the PBZN. The factors are colored in red and green, which indicate significantly negative and positive associations with the surrounding modules, respectively. T, temperature; Sa, salinity; NH4N, ammonia nitrogen; NO3N, nitrate nitrogen; NO2N, nitrite nitrogen; Cu, As, Zn, and Pb, the concentrations of metal factors. ***, *P* < 0.001; **, *P* < 0.01; *, *P* < 0.05.

Since distinct differences in response to the environmental factors were observed in both the PBZNs and the PSZNs, module-eigenvalue analysis was used to further quantify the intrinsic factors driving the PZN structure. There were multiple modules in each regional network that displayed statistically significant correlations with different factors. Two modules were considered to be a preserved module pair between PBZNs and PSZNs if they shared a large proportion of nodes (≥50%) at an individual sampling site (Fig. 4, in which preserved module pairs are colored the same). We then found that the conservation of most modules was derived from the peripheral taxa within each module pair, while the hub taxa composition changed significantly. For example, at site5, the groups of diatoms and gastropods were regarded as hub taxa in the module pair (M1 to M2), but only arthropods were identified as connector hubs in the PBZNs module. Diatoms and *Acetes* were identified as hub taxa in PSZNs, but the hubs became diatoms and *Cirripedia* in the corresponding module of PBZNs at site6 (Fig. 4). Going a step further, we found that although some paired modules exhibited the same significant relationship with environmental factors, the relationships between most modules and environmental factors were altered with larger zooplanktonic body sizes. For example, the paired modules of site5 exhibited significant correlations with the same environmental factors, whereas the M1 in PSZN was significantly correlated with NO_3_-N, and its corresponding module (M5) in PBZN was not significantly associated with any environmental factors at site3. Moreover, modules M1 and M2 in PSZN exhibited significant positive associations with pH at site7, while the paired modules in the PBZN were not sensitive to pH variations. Based on these results, the correlation between network modules and abiotic factors was mainly reflected in the sensitivity of the networked zooplankton taxa to environmental disturbances, and zooplanktonic body size directly shaped the environmental dynamic of the network structure.

## DISCUSSION

In the *Tara Ocean* project, the planktonic species, including both phytoplankton and zooplankton, and their interactions have been investigated at a global scale (20). This and other related large-scale studies have focused on the influences of abiotic factors (i.e., temperature and salinity) on the network structure and topological properties (42, 43) but ignored the influence of body size in their interactions. Also of note, the limitations in the determination of body size by sequencing technology might bring a large gap to such an analysis (17). Thus, morphological investigations might be better for studying the effect of body size in aquatic ecosystems. In the present study, we comprehensively surveyed via microscopy 200 zooplanktonic species with different body sizes, as well as their 307 associated phytoplanktonic species across 251 samples in marine ecosystems along the Chinese coastline. We also examined the effects of zooplanktonic body size on the complexity and stability of phyto-zooplankton networks (PZNs). Notably, although co-occurrence-based statistical approaches are the most widely used method for discerning potential species interrelationships (28, 36, 44, 45), some of the links within PZNs might not represent the real biotic interactions. The biotic interaction, environmental filtering, and dispersal limitation were the three major drivers for the co-occurrence patterns (46). In our analysis, the variation portioning analysis (VPA) revealed that all of the measured coastal variables and distance factors were able to explain only a minor portion of the networked community variations (26.75%) (Fig. S2), indicating that the PZNs were not primarily driven by abiotic environmental filtering. Nevertheless, more evidence is still needed to verify our PZN results in various ecosystems. In general, our study suggested that planktonic investigation and cross-trophic interactions should consider the role of body size in experimental designs.

### Increase in zooplanktonic body size decreased the complexity and stability of PZNs.

Body size, as a key life history trait of plankton, has previously attracted attention in studies of planktonic communities and species interactions, which indicated that body size could affect the dynamics of the aquatic food web (14, 47, 48). As body size increases, the metabolic rate of a species decreases, and the efficiency of energy transfer from lower to higher trophic levels is reduced, resulting in an inverse relationship between body mass and abundance, in which smaller body sizes are present at a higher abundance and density (11, 13, 19, 49). Here, we clearly observed a significantly higher density for smaller zooplankton near the offshore. Notably, zooplankton body size not only determined zooplanktonic density but also strongly affected the structure of PZNs through biotic interactions, which were supported by some complexity indices (network size, links, links per species, and keystone numbers), which significantly decreased with increasing body size (Table 1; Fig. 1). It is generally known that these complexity indexes are significantly positively correlated with network stability (36, 43) and that an increasing number of keystone organisms indicates a more stable network (50). Additionally, the stability indices, robustness, and vulnerability were significantly decreased and increased with the larger zooplankton sizes (Fig. 2 and 3B). Together, these findings indicate that increasing zooplanktonic body size gradually attenuates PZN complexity and stability.

### Decreased stability of PZNs was likely due to niche overlap between zooplanktonic communities with different body sizes.

We think that the lower robustness and higher vulnerability for PZNs with randomly removed zooplanktonic nodes (Fig. 2) might mean that the zooplanktonic nodes were more unstable to networks than were phytoplanktonic nodes, and this could largely be a consequence of zooplanktonic species occupying a more important niche in the coastal ecosystem. As indicated in previous studies, higher-level trophic species in the aquatic food web were the regulators of primary producers, and the phytoplanktonic community was under top-down control (51, 52). Particularly, we demonstrated that the instability of PBZNs was likely due to the niche overlap between differently sized zooplankton in the diatom-dominated plankton ecology of the Chinese coast and that and the larger ones were weak in terms of resource competition. One study has demonstrated that positive relationships in networks represent high niche overlaps (37). In the present study, we found that all of the significant relationships between smaller and bigger zooplankton were positive (Fig. S1b), suggesting strong niche overlaps between these two zooplankton size fractions. Compared with the larger sized zooplankton, the smaller zooplanktonic species are the main grazers of phytoplankton and consume 60 to 70% of the primary production (53). They also had greater dispersal scales because the dispersal efficiency of plankton is determined by water flow and is inversely proportional to body size (14). Similarly, recent studies generated an appreciation for the pivotal role of smaller body sizes in decreasing the maximum ingestion rate of prey and in selecting the optimum prey size (54), with a narrower optimum prey size being observed with zooplanktonic species of smaller sizes (55). This might partially explain the higher robustness values in networks constructed by smaller zooplanktonic species and small diatom-dominated phytoplankton in the coastal ecosystem. These findings indicate that smaller zooplankton have an advantage in niche competition compared to larger zooplankton in the coastal ecology and that this superiority potentially leads to more stable interaction networks between phytoplankton and smaller zooplankton.

### Zooplanktonic body size shapes the stability of subnetworks.

In our study, the stability of dominant phyto-zooplanktonic subnetworks was further analyzed to reveal the potential influence of zooplanktonic sizes in determining coastal plankton ecosystem stability. Diatoms, as particularly important players in the biogeochemical cycle of carbon, are responsible for up to 40% of carbon fixation (56–58), while dinoflagellates are a significant planktonic group that includes some toxic and bloom-forming species (59). Previous studies have shown that the interactions between zooplankton and phytoplankton are pivotal in maintaining the stable states of aquatic ecosystems (60, 61). Here, we noticed that the stability of diatom and dinoflagellate subnetworks varied with zooplanktonic body size, with the smaller and bigger zooplankton tending to construct more stable interaction relationships with diatoms and dinoflagellates, respectively (Fig. S6). Because predator-prey interactions constitute the majority of planktonic protistan interactions (62), the differences in the stability of the dominant phytoplankton subnetworks might provide new insights regarding the biological control of algal blooms. Based on the above research, we hypothesize that zooplankton with smaller sizes might contribute to the control of the less toxic algal blooms near the Chinese offshore area, which are dominated by diatoms, while larger-sized zooplankton might be better for the prevention of the more toxic blooms by dinoflagellates. Hence, our findings reinforce the point that zooplanktonic body size is an important factor in determining the stable relationship interactions of different dominant phytoplankton and suggest that specific biological treatment strategies for algal blooms formed by different phytoplankton taxa should involve the consideration of the size of the planktonic predators employed.

### Zooplanktonic body size determined the effects of abiotic properties on PZNs.

Environmental disturbances could substantially decrease the persistence and resilience of communities by reducing the stability of microbial interaction networks (43, 63). Recent studies have shown that the co-occurrence network stability of microeukaryotic plankton can be shaped by salinity and that temperature was negatively associated with robustness in the subtropical urban reservoir and global ocean (42, 43). In this study, we found that the phytoplanktonic taxa in PSZNs and PBZNs were only sensitive to metal concentrations near the offshore and that body size could significantly alter the sensitivity of the zooplanktonic community to abiotic properties (Table 2). As shown in previous studies, microbial stability is mainly affected by specific groups within the network that initially respond to environmental variations (21), and higher trophic level organisms were more sensitive to environmental changes, such as temperature (64, 65). Our results showed that while the phytoplanktonic composition was consistent in most paired modules, the zooplanktonic composition changed significantly with body size, and the correlations between the vast majority of paired modules and environmental factors were significantly changed (Fig. 4). Environmental factors (including water traits and metal concentrations) mainly shaped the PZNs through zooplankton, and no factor had an indirect influence on the PZNs through phytoplankton (Fig. S7). Our results suggest that the abiotic environmental changes through the effects on zooplanktonic species influence the PZNs, which could further impact the density and compositions of the phytoplanktonic communities in aquatic ecosystems.

### Conclusions.

In the present study, our findings serve to unravel the importance of zooplanktonic size in determining the complexity and stability of planktonic cross-trophic networks. In general, our findings revealed that zooplankton play a decisive role in maintaining co-occurrence networks and that more complex and stable PZNs are possible with smaller zooplanktonic sizes. Particularly, by identifying the variety of relationships between differently sized zooplankton and dominant phytoplankton taxa, we elucidated the role of zooplanktonic body size in determining specific subnetwork stability. Furthermore, the variation of zooplankton size not only changed the sensitivity of the planktonic species to environmental disturbances but also directly shaped the relationships between environmental factors and PZNs. Overall, this study provided new insights into the effect of zooplanktonic size on phyto-zooplanktonic co-occurrence patterns and demonstrated a critical role of body size in the complex biotic associations in aquatic ecosystems.

## MATERIALS AND METHODS

### Sampling and data collection.

We previously compiled a data set that was analyzed for plankton composition and the spatial patterns of phytoplankton and zooplankton interactions. Water samples were collected from 251 locations along the Chinese coastline from August to October 2017 (41), and these were visualized using ArcMap 10.3 (Fig. S1A). Planktonic samples were collected using a plankton net via a vertical tow from 2 m above the bottom to the surface with a speed of 0.5m/s, with type I, II, and III nets being used to collect bigger (~500 μm) size zooplankton, smaller (~160 μm) size zooplankton, and phytoplankton, respectively (Chinese National Standard: GB 17678.7-2007). The phytoplanktonic and zooplanktonic samples were preserved immediately in 5% formaldehyde. The samples that needed individual identification in the laboratory were fixed with 2% glutaraldehyde and were microscopically identified at the lowest allowable taxonomic level (Chinese National Standard: GB/T 12763.6-2007) (66). Then, each taxonomic name was verified based on the World Register of Marine Species (WoRMS, http://www.marinespecies.org/). The three types of nets caught 240, 282, and 307 planktonic species, respectively. In order to exclude the influence of zooplanktonic composition on the network analysis, we matched small and big size zooplankton species and only retained the zooplankton species caught by both type I and type II nets. In addition, the zooplankton density was calculated by 1 ind/L. The seawater temperature data were obtained from the National Marine Data Center (NMDC; http://mds.nmdis.org.cn). The pH and salinity were determined using a pH meter and a multiparameter sensor (YSI 6600). The inorganic nutrients (NO_3_-N, NO_2_-N, and NH_4_-N) were analyzed based on published protocols (67). An inductively coupled plasma mass spectrometry and atomic fluorescence spectrometer (AFS-920) was used to analyze the concentrations of dissolved heavy metals.

### Network construction and networked community analyses.

Feng et al. set up a workflow, named the integrated Network Analysis Pipeline (iNAP, http://mem.rcees.ac.cn:8081), to find the relationships between interdomain taxonomic groups by using the SparCC approach (29, 68, 69). This method provided technical support for analyzing cross-trophic associations, such as those between phytoplankton and zooplankton (21). To investigate the dynamics of plankton interaction patterns with zooplankton size variation, we constructed cross-trophic plankton interaction networks called phyto-zooplankton networks (PZNs) by calculating the pairwise correlation between phytoplankton and zooplankton in the iNAP.

Taken together, all of the PZNs involved all zooplankton (smaller and bigger zooplankton) and phytoplankton present in 251 samples. In order to further investigate the variation patterns of PZNs in relation to zooplankton size, an additional 24 regional PZNs were constructed along the latitude, half of which were for phytoplankton-bigger zooplankton interaction networks (PBZNs) and half of which were for phytoplankton-smaller zooplankton interaction networks (PSZNs). Noncorrelated associations in the matrix were filtered under the threshold value of 0.3, and a statistical significance threshold of 0.05 was used to generate bipartite network. The constructed networks were visualized in Gephi 0.9.2 and Cytoscape 3.7.2. The PZNs topological indices, such as the nodes, links, links per species, cluster coefficient, linkage density, and weighted connectance, were calculated using iNAP to characterize the structure of the PZNs (69). To determine the randomness of the PBZN and PSZN, 1,000 bipartite networks were randomly generated by using the Maslov-Sneppen procedure (70), and these were compared with the observed network based on the one-sample Student’s *t* test. Based on the within-module connectivity (*Zi*) and among-module connectivity (*Pi*) performed in iNAP, the network nodes were classified into four categories: module hubs (*Zi *> 2.5 and *Pi *≤ 0.62), network hubs (*Zi *> 2.5 and *Pi *> 0.62), connectors (*Zi *≤ 2.5 and *Pi *> 0.62), and peripherals (*Zi *≤ 2.5 and *Pi *≤ 0.62) (71). Except for the peripherals, all of the types of nodes are referred to as network keystones (25). All statistical analyses were performed in R (v3.6.1, http://www.r-project.org). The statistical significance of the networked zooplanktonic density was evaluated using the Kruskal-Wallis test via the *geom_signif* function (R package: ggsignif) (72). Nonmetric multidimensional scaling (NMDS) analysis was performed based on the Bray-Curtis distances via *metaMDS* (R package: vegan) to visualize the variation in the networked zooplanktonic community composition. Three complementary methods, PERMANOVA, ANOSIM, and MRPP, based on the Bray-Curtis distances were performed to examine the dissimilarities in the zooplanktonic community (R package: vegan) (73). A PCNM analysis was utilized to generate the spatial distance variables via *pcnm* (R package: vegan). A CCA was used to assess the effects of water traits, spatial distance, and other environmental factors on the networked communities, and the contributions of these variables were examined via a VPA.

### Network analyses.

**(i) Linking abiotic factors to the network structure**. Mantel tests were used to calculate the correlation between the connectivity and the significance of nodes to distinguish the impacts of environmental factors on the network structure. To detect the modules’ responses to environmental changes, the relationships among module-based eigenvalues and environmental factors were calculated and visualized using the ‘pheatmap’ package in R (74). Structural equation modeling was utilized to evaluate the direct links between the environmental factors, plankton community composition, and network structure, the structural equation model was fitted using the “lavaan” package in R (75). The χ^2^ test, the root mean square error of approximation (RMSEA), and the comparative fit index (CFI) were used to judge the fit of the model (76). Water traits (temperature, salinity, and dissolved oxygen [DO]), metal factors, and N elements (nitrate nitrogen, nitrite nitrogen, and ammonia nitrogen) were represented by the PC1 scores. The planktonic composition and the network structure were represented by the first component of the NMDS analysis and the module eigenvalue results, respectively.

**(ii) Network stability analyses.**. In order to characterize the network stability, robustness and vulnerability were calculated. Robustness was defined as the proportion of nodes remaining in the PZNs after the random removal of nodes and was calculated as the abundance unweighted mean interaction strength of nodes in the bipartite plankton interaction networks (77). In order to determine the significance of robustness for the observed PZNs, the robustness of removing zooplanktonic and phytoplanktonic nodes, respectively, was calculated for 1,000 random networks generated using the Maslov-Sneppen procedure (70), and the significance was measured using a one-sample Student's *t* test. In the present report, for the bipartite network, we calculated the remaining nodes for two scenarios, by the removal of either phytoplankton or zooplankton nodes, to discern the contribution to the stability of the PZNs by species from the two different trophic levels. The vulnerability of each node measures the relative contribution of the node to the global efficiency, and the maximal vulnerability of nodes indicates the networked vulnerability, which are calculated as

$$\text{max}(\frac{E-E_{i}}{E})\;\text{and }E=\frac{1}{n\left(n-1\right)}\underset{i\neq j}{\sum }\frac{1}{d(i,j)}$$

where *E* and *E_i_* are the global efficiency and the global efficiency after removing node *i*, respectively, and *d_ij_* is the shortest path between node *i* and node *j* (25).

### Data availability statement.

The planktonic species tables are available from our own webpage, http://mem.rcees.ac.cn/index.php/download/.
