# Supplementary figures and images for: The Stability of Phyto-Zooplanktonic Networks Varied with Zooplanktonic Sizes in Chinese Coastal Ecosystem
Source: mSystems. 2022 Oct 6;7(5):e00821-22. doi: 10.1128/msystems.00821-22 (PMC9599403; doi:10.1128/msystems.00821-22)

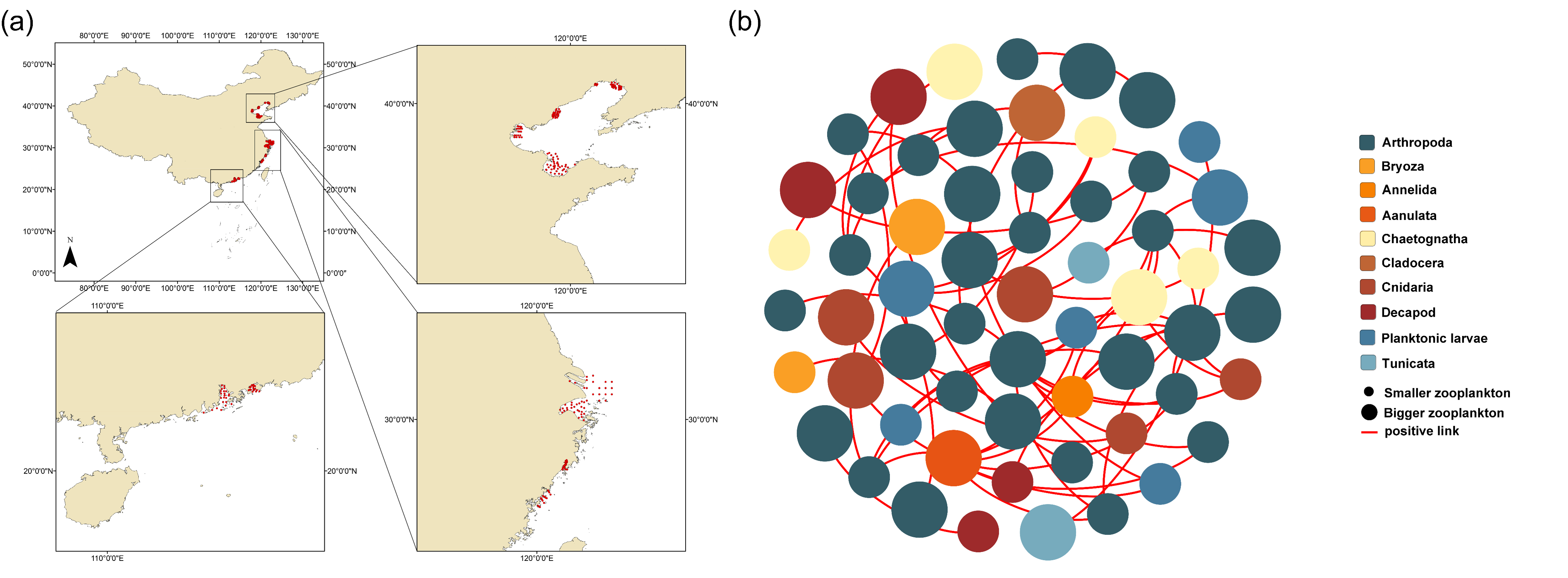

Supplement: FIG S1 [file msystems.00821-22-s0001.tif]

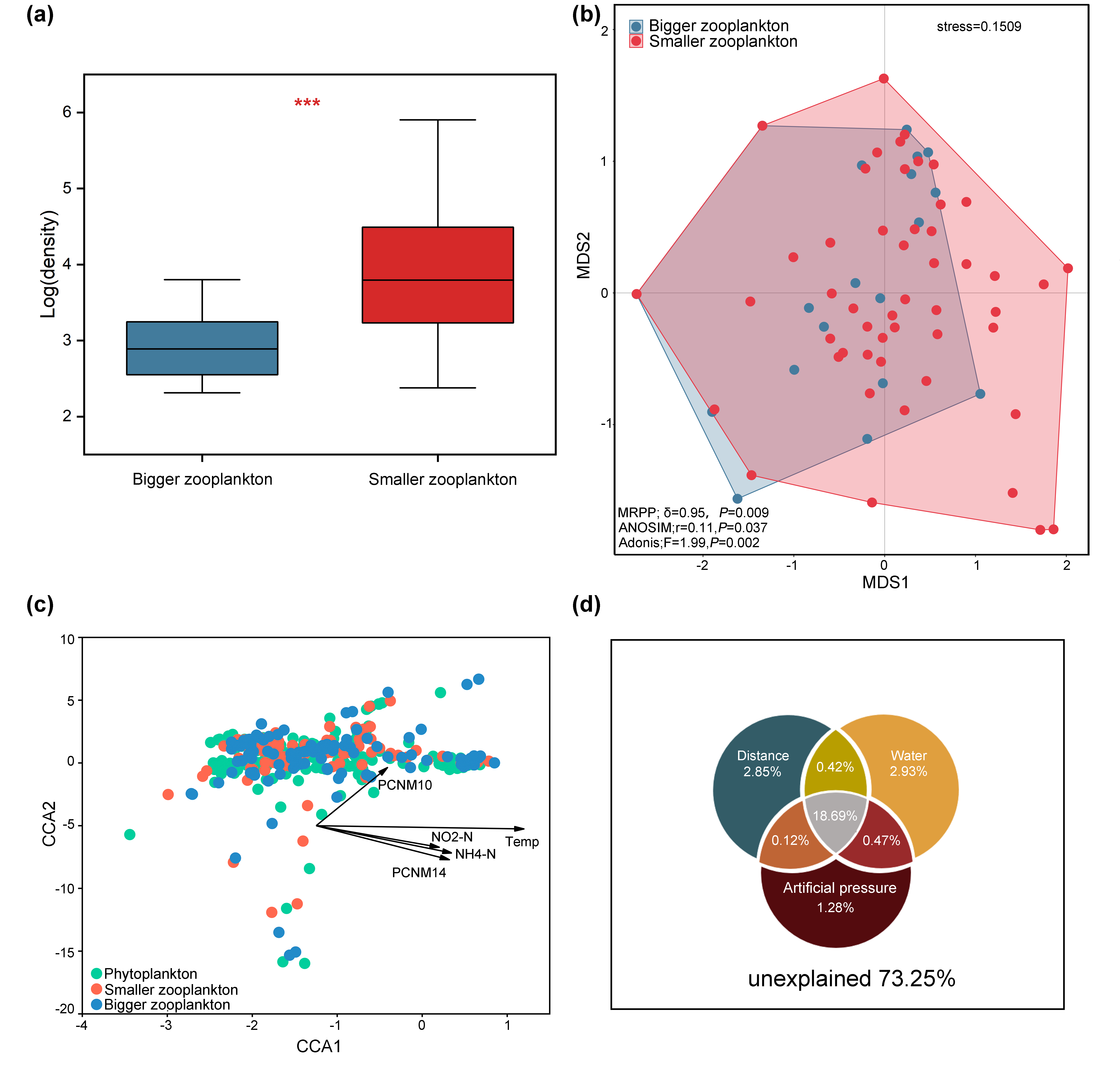

Supplement: FIG S2 [file msystems.00821-22-s0002.tif]

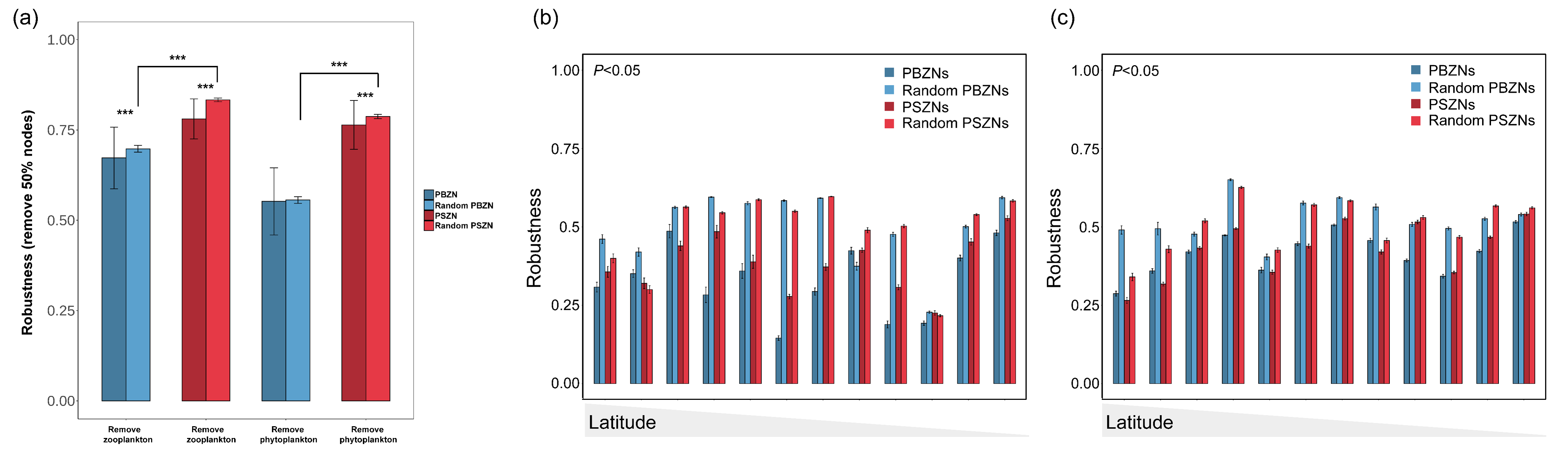

Supplement: FIG S3 [file msystems.00821-22-s0003.tif]

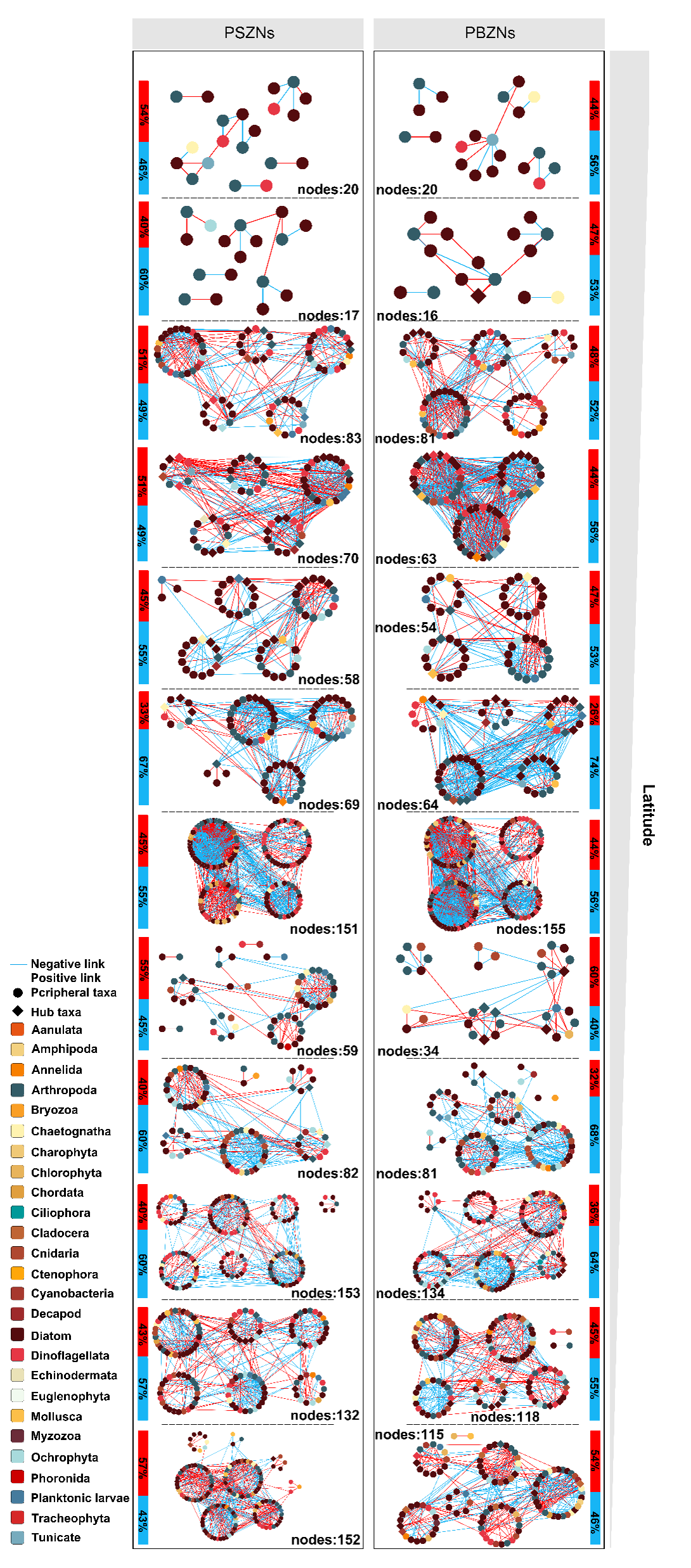

Supplement: FIG S4 [file msystems.00821-22-s0004.tif]

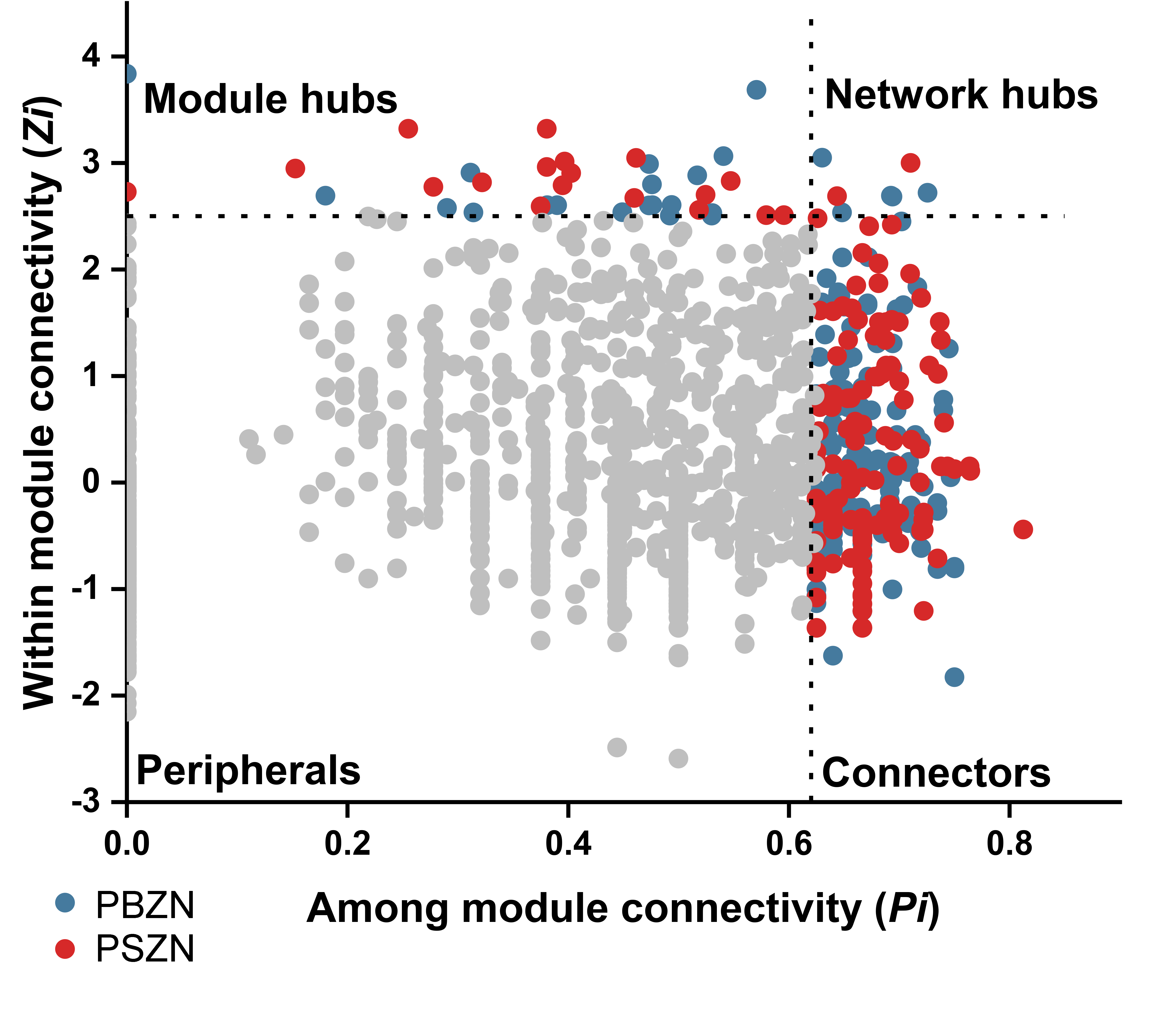

Supplement: FIG S5 [file msystems.00821-22-s0005.tif]

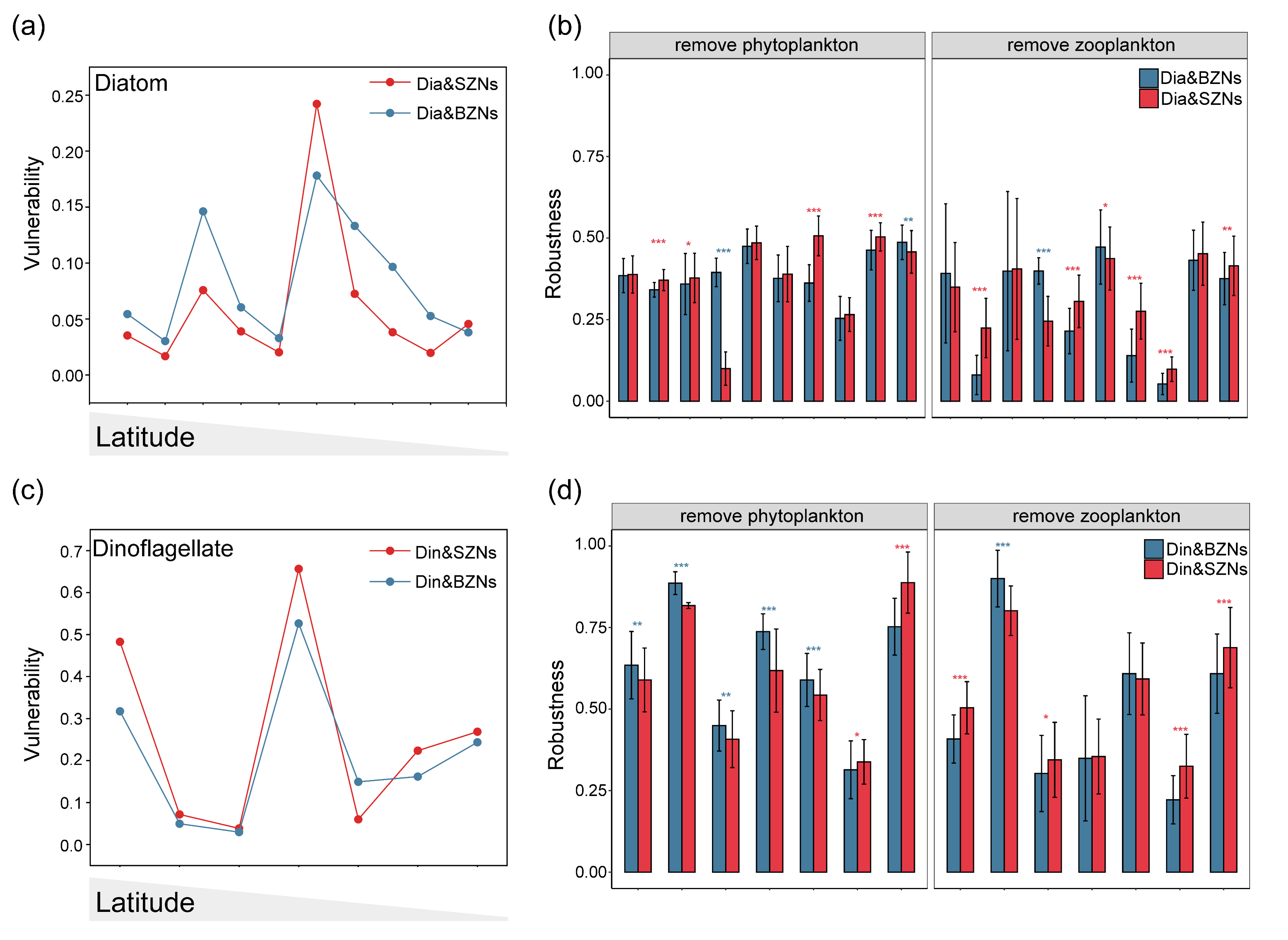

Supplement: FIG S6 [file msystems.00821-22-s0006.tif]

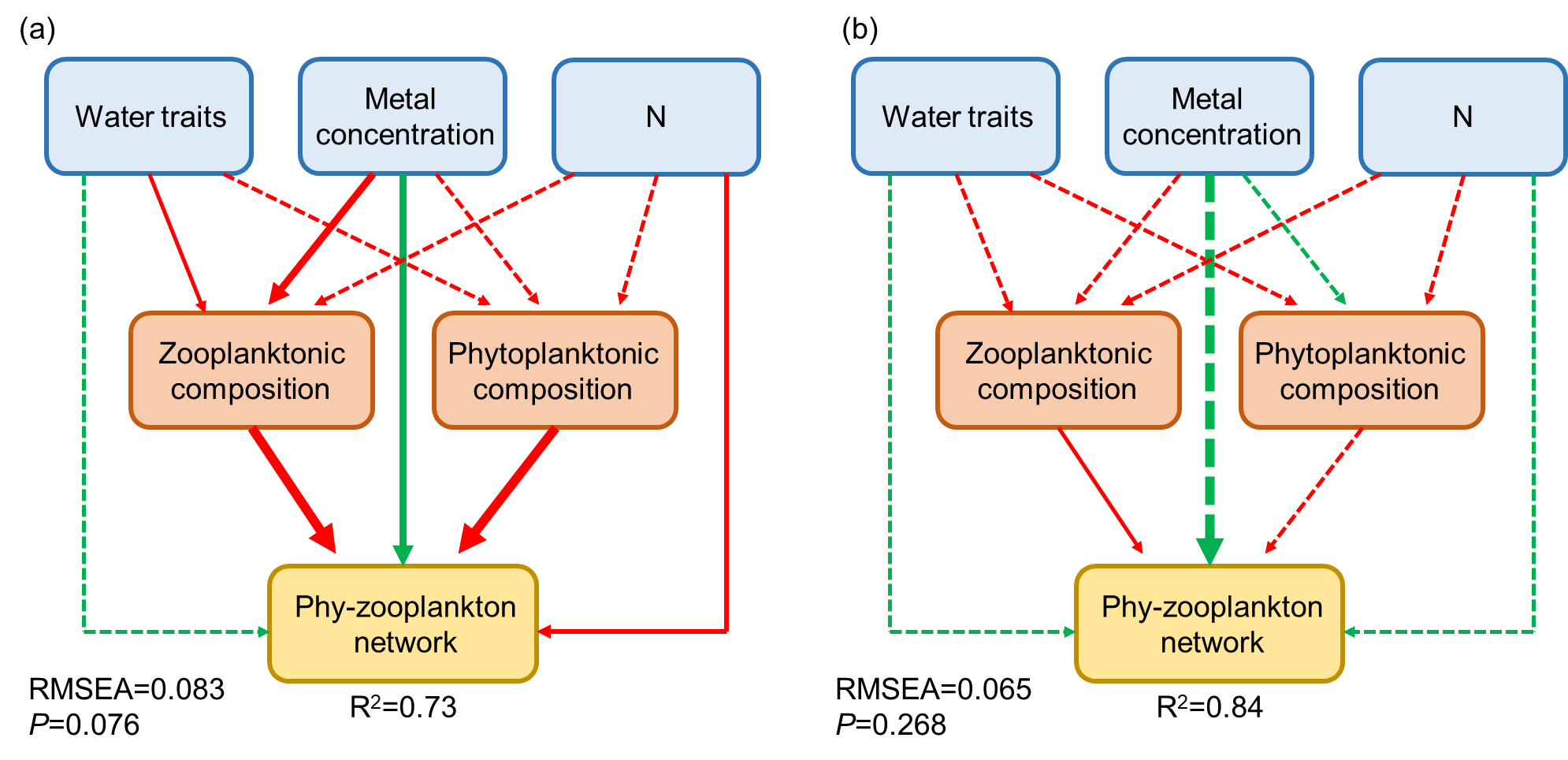

Supplement: FIG S7 [file msystems.00821-22-s0007.tif]
