# Supplementary material for: The Stability of Phyto-Zooplanktonic Networks Varied with Zooplanktonic Sizes in Chinese Coastal Ecosystem
Source: mSystems. 2022 Oct 6;7(5):e00821-22. doi: 10.1128/msystems.00821-22 (PMC9599403; doi:10.1128/msystems.00821-22)
Supplement: TABLE S1 [file msystems.00821-22-s0008.docx]

**Table S1.** ANOVA of environmental factors and distance correlated with networked planktonic beta diversity. The significance is shown by number in bold.

|  | F | *P* |
| --- | --- | --- |
| PCNM2 | 2.03 | 0.059 |
| PCNM3 | 1.71 | 0.096 |
| PCNM4 | 1.64 | 0.154 |
| PCNM6 | 1.49 | 0.186 |
| PCNM8 | 2.00 | 0.140 |
| PCNM10 | 2.24 | **0.021** |
| PCNM11 | 2.23 | 0.129 |
| PCNM12 | 1.92 | 0.084 |
| PCNM14 | 3.04 | **0.001** |
| Temperature | 6.24 | **0.001** |
| Zn | 1.77 | 0.063 |
| Pb | 2.07 | 0.135 |
| As | 3.04 | 0.063 |
| NO2N | 2.48 | **0.002** |
| NH4N | 2.89 | **0.013** |
| Salinity | 1.73 | 0.129 |
| Chlorophylla | 1.08 | 0.269 |
| COD | 1.78 | 0.081 |
| DO | 1.82 | 0.09 |
