# Supplementary material for: The Stability of Phyto-Zooplanktonic Networks Varied with Zooplanktonic Sizes in Chinese Coastal Ecosystem
Source: mSystems. 2022 Oct 6;7(5):e00821-22. doi: 10.1128/msystems.00821-22 (PMC9599403; doi:10.1128/msystems.00821-22)
Supplement: TABLE S2 [file msystems.00821-22-s0009.docx]

**Table S2**. Topological properties of the PZNs with different sized zooplankton along the Chines coastline.

|  |  | Network size | Links | Linkage density | Links per species |
| --- | --- | --- | --- | --- | --- |
| Site1 | PBZN | 20 | 16 | 2.688 | 0.8 |
|  | PSZN | 20 | 15 | 2 | 0.75 |
| Site2 | PBZN | 16 | 15 | 2.8 | 0.938 |
|  | PSZN | 17 | 13 | 2.077 | 0.765 |
| Site3 | PBZN | 81 | 231 | 9.883 | 2.852 |
|  | PSZN | 83 | 219 | 8.480 | 2.639 |
| Site4 | PBZN | 63 | 488 | 19.293 | 7.746 |
|  | PSZN | 70 | 302 | 13.546 | 4.314 |
| Site5 | PBZN | 54 | 126 | 8.437 | 2.333 |
|  | PSZN | 58 | 141 | 8.284 | 2.431 |
| Site6 | PBZN | 64 | 315 | 14.438 | 4.922 |
|  | PSZN | 69 | 245 | 10.776 | 3.551 |
| Site7 | PBZN | 155 | 980 | 23.090 | 6.323 |
|  | PSZN | 151 | 847 | 20.955 | 5.609 |
| Site8 | PBZN | 34 | 48 | 4.625 | 1.412 |
|  | PSZN | 59 | 108 | 6.435 | 1.831 |
| Site9 | PBZN | 81 | 212 | 9.434 | 2.617 |
|  | PSZN | 82 | 220 | 8.768 | 2.683 |
| Site10 | PBZN | 134 | 379 | 10.243 | 2.828 |
|  | PSZN | 153 | 379 | 8.850 | 2.477 |
| Site11 | PBZN | 118 | 328 | 8.817 | 2.779 |
|  | PSZN | 132 | 380 | 8.184 | 2.879 |
| Site12 | PBZN | 115 | 318 | 9.242 | 2.765 |
|  | PSZN | 152 | 547 | 13.080 | 3.599 |
