# Supplementary material for: The Stability of Phyto-Zooplanktonic Networks Varied with Zooplanktonic Sizes in Chinese Coastal Ecosystem
Source: mSystems. 2022 Oct 6;7(5):e00821-22. doi: 10.1128/msystems.00821-22 (PMC9599403; doi:10.1128/msystems.00821-22)
Supplement: TABLE S3 [file msystems.00821-22-s0010.docx]

**Table S3.** Mantel tests on the significance of environmental factors and planktonic community composition. The significance is shown by numbers in bold

|  | Phytoplankton | | Smaller zooplankton | | Bigger zooplankton | |
| --- | --- | --- | --- | --- | --- | --- |
|  | r | *P* | r | *P* | r | *P* |
| Temperature | 0.326 | **0.001** | 0.361 | **0.001** | 0.347 | **0.001** |
| pH | 0.210 | **0.001** | 0.244 | **0.001** | 0.213 | **0.001** |
| Salinity | 0.256 | **0.001** | 0.319 | **0.001** | 0.235 | **0.001** |
| Hg | 0.003 | 0.435 | 0.113 | **0.023** | 0.130 | **0.001** |
| Pb | 0.022 | 0.282 | 0.095 | **0.036** | 0.111 | **0.002** |
| As | 0.019 | 0.291 | 0.118 | **0.015** | 0.138 | **0.001** |
| Cu | 0.021 | 0.278 | 0.086 | 0.073 | 0.101 | **0.003** |
| Zn | 0.004 | 0.445 | 0.118 | **0.019** | 0.136 | **0.001** |
| NO_3_-N | 0.250 | **0.001** | 0.290 | **0.001** | 0.205 | **0.001** |
| NO_2_-N | -0.026 | 0.727 | -0.081 | 0.954 | 0.029 | 0.175 |
| NH_4_-N | 0.053 | 0.116 | 0.021 | 0.319 | 0.077 | **0.018** |
